# Cancer risk in waterpipe smokers: a meta-analysis

**DOI:** 10.1007/s00038-016-0856-2

**Published:** 2016-07-15

**Authors:** Ravinder Mamtani, Sohaila Cheema, Javaid Sheikh, Ahmad Al Mulla, Albert Lowenfels, Patrick Maisonneuve

**Affiliations:** 1 Department of Global and Public Health, Weill Cornell Medicine-Qatar, Doha, Qatar; 2 Dean’s Office, Weill Cornell Medicine-Qatar, Doha, Qatar; 3 Smoking Cessation Program, Hamad Medical Corporation, Doha, Qatar; 4 Department of Surgery, New York Medical College, Valhalla, New York, USA; 5 Division of Epidemiology and Biostatistics, European Institute of Oncology, Milan, Italy

**Keywords:** Waterpipe, Shisha, Hubble bubble, Smoking, Cancer, Meta-analysis

## Abstract

**Objectives:**

To quantify by meta-analysis the relationship between waterpipe smoking and cancer, including cancer of the head and neck, esophagus, stomach, lung and bladder.

**Methods:**

We performed a systematic literature search to identify relevant studies, scored their quality, used fixed and random-effect models to estimate summary relative risks (SRR), evaluated heterogeneity and publication bias.

**Results:**

We retrieved information from 28 published reports. Considering only highquality studies, waterpipe smoking was associated with increased risk of head and neck cancer (SRR 2.97; 95 % CI 2.26–3.90), esophageal cancer (1.84; 1.42–2.38) and lung cancer (2.22; 1.24–3.97), with no evidence of heterogeneity or publication bias. Increased risk was also observed for stomach and bladder cancer but based mainly on poor-quality studies. For colorectum, liver and for all sites combined risk estimates were elevated, but there were insufficient reports to perform a meta-analysis.

**Conclusions:**

Contrary to the perception of the relative safety of waterpipe smoking, this meta-analysis provides quantitative estimates of its association with cancers of the head and neck, esophagus and lung. The scarcity and limited quality of available reports point out the need for larger carefully designed studies in well-defined populations.

## Introduction

For several hundred years, waterpipe smoking, sometimes known as “Shisha” or “Hubble bubble” smoking, has been a common form of smoking in the Middle East. It is especially popular with younger smokers and rapidly becoming popular in other regions (Maziak 2011, 2015). With increasing restrictions on cigarette smoking in public venues, there has been a rapid and potentially dangerous rise in hookah bars where patrons can smoke in an unregulated environment. These bars are often located near colleges or universities so that they attract younger individuals (Maziak et al. 2015).

The reasons for its growing popularity include: (1) the perception that this form of tobacco exposure is much safer than cigarette smoking, since tobacco smoke is filtered through water (Aljarrah et al. 2009); (2) the cost of waterpipe smoking is lower than cigarette smoking, which in most countries is heavily taxed (Nakkash et al. 2011); (3) waterpipe smoking is often a social experience in a dedicated venue where several persons share the same apparatus; hookah bars are often excluded from regulations pertaining to indoor smoking (Tee et al. 2015).

The negative effects of cigarette smoking on health have been known since the middle of the 20th century and we now know that approximately half of lifetime smokers will die from smoking related diseases, with cancer accounting for approximately half of these deaths (US Department of Health and Human Services 2014). The health effects of waterpipe smoking are less well known, especially with respect to the risk of cancer (El-Zaatari et al. 2015; Maziak 2012).

The aim of this study was to employ meta-analytic techniques to update and quantify existing reports of the risk of cancer associated with waterpipe smoking.

## Methods

### Search strategy, inclusion criteria, and data abstraction

#### Waterpipe and cancer (meta-analysis)

We performed a systematic literature search using PubMed, without language or other restrictions, looking for papers referring to the use of waterpipe, also known as shisha, narghile, arghileh, hubble-bubble or hookah and cancer. We also used other databases (Web of Science™, Google Scholar). In particular, we retrieved from Web of Science papers citing any of the study reports previously identified or any of the major reviews on the topic. We also scrutinized references of relevant papers. Finally, we searched PubMed in a more empirical manner for observational studies on the association between tobacco smoking and cancer conducted in Middle East countries, where waterpipe is a common form of smoking (i.e., cancer AND smoking AND Iran). Only reports fulfilling the following inclusion criteria were included in the meta-analysis.

- One reviewer (PM) was involved in the appraisal of papers identified through the main PubMed search and extracted data necessary for the study in a pre-defined spreadsheet. A second reviewer (ABL) controlled the suitability of the studies identified by the first reviewer and verified the accuracy of the data extracted.
- Studies that contained the minimum information necessary to estimate the relative risk (RR) of any form of cancer associated with waterpipe smoking and a corresponding measure of uncertainty [i.e. 95 % confidence interval (CI), standard error, variance, or *P* value of the significance of the estimate].
- Case–control and cohort studies, published as original articles.
- Studies that were independent. In case of multiple reports on the same population or subpopulation, we considered the estimates from the most recent or most informative report.

When available, we used adjusted risk estimates and those based on population-based controls. For reports presenting only tabular data, we calculated crude relative risks and corresponding 95 % confidence intervals. We used the Newcastle Ottawa scale (NOS) to assess the quality of the included studies (Wells et al. 2009). For case–control studies, a maximum of four points were given for the selection of cases and controls, two points for the comparability of cases and controls on the basis of the design or analysis, and four points for the ascertainment of exposure (waterpipe smoking). For cohort studies, a maximum of four points was given for the selection, two points for the comparability, and three points for the ascertainment of outcome. We considered that control/adjustment for other form of tobacco smoking (or restriction of the analysis to exclusive waterpipe smokers) was the most important factor for the comparability.

**Fig. 1:**
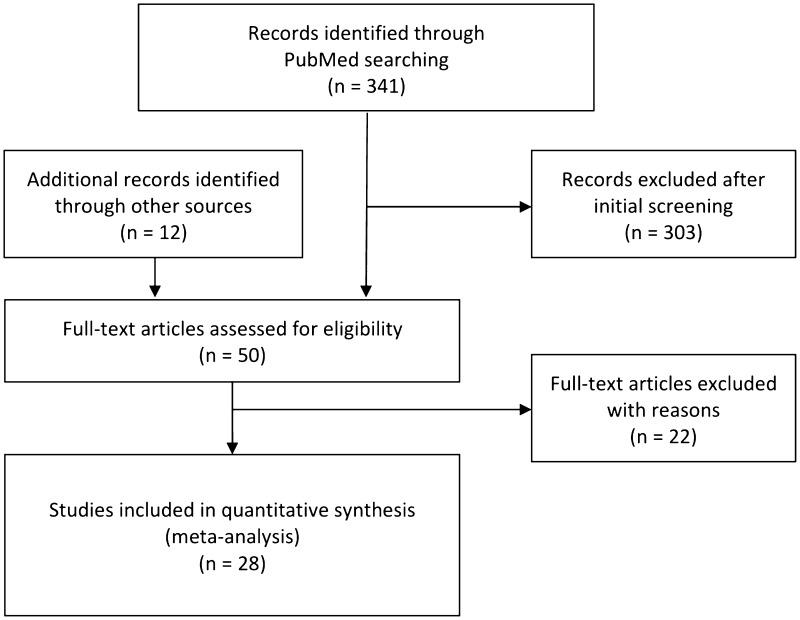
Flow diagram: eligibility assessment of potential studies on waterpipe smoking and cancer identified from literature search or from other sources, for inclusion in the meta-analysis

Overall, 341 references published up to 23 June 2015 were retrieved from the following PubMed search query: ((Waterpipe OR shisha OR narghile OR arghileh OR hubble-bubble OR hookah) AND (“neoplasms”[MeSH Terms] OR “neoplasms” OR “cancer”)) OR ((Iran OR Iraq OR Egypt OR Oman OR Qatar OR Jordan OR Syria OR Libya OR Yemen OR Tunisia OR Saudi Arabia OR Pakistan OR Kashmir) AND (“neoplasms”[MeSH Terms] OR “neoplasms” OR “cancer”) AND (“case–control studies”[MeSH Terms] or “case–control” or “case control”) AND (“smoking” OR “tobacco”)). Titles and abstracts available in PubMed were evaluated and full text of 38 study reports was obtained. Full texts of 12 additional study reports identified from other sources (mostly citations in published reviews on the topic) were also evaluated for inclusion in the meta-analysis. Twenty-two were excluded: eight were lacking a control group, four contained no original data, three referred to non-malignant lesions, four presented data included in other reports and in three studies no distinction between waterpipe and other forms tobacco smoking was made. Twenty-eight studies satisfied the eligibility criteria and were included in the synthesis (Fig. 1).

### Statistical analysis

Since only case–control studies were identified for the meta-analysis, we used odds ratios as a measure of the association between waterpipe smoking and the various forms of cancer. In absence of heterogeneity of the risk estimates from individual studies, we used fixed effect models to estimate summary relative risks. In the presence of heterogeneity, we used random effects models to account for this heterogeneity and to provide more conservative risk estimates, Homogeneity of effects across studies was assessed using the *I* ^2^ statistic (Higgins et al. 2002), which represents the percentage of total variation across studies that is attributable to heterogeneity rather than chance. When several risk estimates were present in a single study (i.e., separate estimates for men and for women), we adjusted the summary risk estimates for intra-study (or within-study) correlation (van Houwelingen et al. 2002). Publication bias was graphically evaluated by funnel plots and quantified by the test developed by Macaskill et al. (2001), obtained by a regression of log (OR) on the sample size, weighted by the inverse of the variance. Meta-analysis was performed using Review Manager software (RevMan) version 5.3.5 (Copenhagen: The Nordic Cochrane Centre, The Cochrane Collaboration, 2014) and SAS software version 9.2 (Cary, NC).

## Results

Table 1 contains detailed information about the 28 individual studies included in this report. Four studies dealt with head and neck cancer, six with esophageal cancer, four with stomach cancer, six with lung cancer, five with bladder cancer, and three studies contain data on two other types of cancer, and one focused on all cancer deaths.

**Table 1:** Characteristics of studies on the association between waterpipe smoking and cancer

References	Study, year Region, country	Cases/controls		OR (95 % CI)	Adjustments	Controls characteristics	Study quality (NOS)			
							Selection	Comparability	Exposure	NOS
Head and neck										
Jaferey (1976)	CC, 1967–1972 Karachi, Pakistan		1192/3562	3.10 (2.23–4.29)	Derived from frequencies, adjusted for sex, cigarettes and bidi smoking	Healthy controls matched for age, sex and place of birth	●●●●	●●	○○●○	7
Feng (2009)	CC, 2002–2005 Algeria, Morocco, Tunisia		636/615	0.49 (0.20–1.23)	Matched analysis adjusted for age and socio-economical status	Hospital controls or friends and family members matched for centre age, sex and childhood household type (urban/rural)	●●○●	○●	○○●●	6
Khlifi (2013)	CC, 2007–2009 Sfax, Tunisia		169/351	2.73 (1.65–4.41)	Adjusted for potential risk factors including smoking	Population based controls	●●●●	●●	○○●○	7
Quadri (2015)	CC, 2014 Jazan, Saudi Arabia		48/96	4.20 (1.32–13.3)	Adjusted for other forms of smoking	Hospital controls matched on sex, age (±5 years) and location	●●○●	●●	○○●○	6
Esophagus										
Cook-Mozaffari (1979)	CC, 1975–1976 Caspian littoral, Iran		217/343 M 127/254 F	1.25 (0.74–2.08) 1.15 (0.43–2.94)	Matched analysis	Matched on sex, age (±5 years) and residence	○●●●	○●	○○○○	4
Nasrollahzadeh (2008)	CC, 2003–2007 Golestan, Iran		300/571	1.69 (0.76–3.77)^a^	Matched analysis adjusted for education and ethnicity	Matched on sex, age (±2 years) and residence	●●●●	●●	○○●○	7
Malik (2010)	CC, 2006–2008 Kashmir Valley, India		135/195	21.4 (11.6–39.5)	Adjusted for age and sex	Routine check-up controls matched for age and sex	●●○●	○●	○○●○	5
Khan (2011)	CC, 2008 Kashmir Valley, India		100/100	9.11 (4.44–18.7)	Derived from frequencies (66/28/6 vs. 15/58/27)		●●●○●	○○	○○●○	4
Dar (2012)	CC, 2008–2012 Kashmir Valley, India		702/1663	1.85 (1.41–2.44)	Adjusted for age, ethnicity, religion, residence, education, cigarette, nass, bidi, cannabis, gutka, alcohol, fruit and vegetables intake	Hospital-based controls matched on sex, age (±5 years) and residence	●●○●	●●	○○●●	7
Stomach										
Pourfarzi (2009)	CC, 2003–2005 Ardabil, Iran		217/394	1.14 (0.29–4.42)	Adjusted for gender, age group, education, family history of GC, various food habits and H. Pylori infection	Community controls matched on sex, age (±5 years) and residence	●●●●	●●	○○●○	7
Shakeri (2013)	CC, 2004–2011 Golestan, Iran		309/613	1.10 (0.30–3.30)	Adjusted for age, ethnicity, education, fruit and vegetable consumption, socioeconomic status, opium, cigarette and nass use.	Healthy controls matched on sex, age (±5 years) and residence	●●●●	●●	○○●○	7
Karajibani (2014)	CC, 2011–2012 Zahedan, Iran		46/46	4.50 (1.17–17.4)	Derived from frequencies (11/46 vs. 3/46)	Matched on sex, age, job, economic status	●●○●	○○	○○●○	4
Sadjadi (2014)	Cohort of Helicobacter pylori infected subjects; Ardabil, Iran		36/928	3.44 (1.66–7.11)	Adjusted for age, sex, family history of cancer, cigarettes smoking, opium, alcohol, fruit and vegetables and salt.	Average follow-up of 10 years	●●●●	●●	●●●	9
Lung										
Qiao (1989)	CC, 1967–1984 Yunnan, China		107/107	1.90 (0.40–9.40)*	Adjusted for age		●●●●	●●	○○●○	7
Lubin (1992)	CC, 1984–1988 Yunnan, China		427/1011	1.78 (0.80–4.20)*	Adjusted for age, residence, type of respondent and years of work	Matched on age (±5 years)	●●●●	●●	○○●○	7
Hsairi (1993)	CC, 1988–1989 Tunis, Tunisia		110/110	5.70 (1.20–7.60)	Adjusted for age, sex, cigarette consumption and cannabis use	Matched on sex, age (±5 years) and cigarettes (±5 cig/day)	●●●●	●●	○○●○	7
Gupta (2001)	CC, 1995–1997 Chandigarh, India		265/525	1.94 (0.85–4.44)	Adjusted for age and education	Hospital controls matched on sex and age	●●○●	○●	○○●○	5
Koul (2011)	CC, 2005–2006 Kashmir Valley, India		251/500	5.83 (3.95–8.60)*	Crude Odds Ratio	Matched on age and residence	●●○●	●●	○○●○	6
Aoun (2013)	CC, 2012 Beirut, Lebanon		50/100	6.00 (1.78–20.3)	Crude Odds Ratio	Hospital controls and visitors matched on sex	●●○●	○○	○○●○	4
Bladder										
Makhyoun (1974)	CC, 1966–1971 Alexandria and Tanta, Egypt		Bilharzial 278/278 Ctr 87/87	1.08 (0.77–1.51) 0.89 (0.45–1.76)	Derived from frequencies (113/278 vs. 108/278) (21/87 vs. 23/87)	Hospital controls matched for antecedent bilharziasis infection, residence and occupation	●●○●	○○	○○●○	4
Bedwani (1997)	CC, 1994–1996 Alexandria, Egypt		151/157	0.80 (0.20–4.00)	Adjusted for age, education, housing, history of schistosomiasis, high risk occupation and tobacco smoking	Hospital controls with acute non-neoplastic, non-urinary tract, non smoking-related condition	●●○●	●●	○○●○	6
Wolpert (2010)	CC, N/a Cairo, Minia, Assiut, Egypt		239/540	1.70 (0.38–7.67)	Derived from frequencies (3/239 vs. 4/540)	Matched on sex, age (±5 years) and residence	●●●●	○●	○○●○	6
Feki-Tounsi (2013)	CC, 2007–2010 Sfax, Tunisia		125/204	1.32 (0.35–5.00)	Derived from frequencies (4/125 vs. 5/204)	Hospital c ontrols consulting for benign disease	●●○●	○○	○○●○	4
Amr (2014)	CC, 2006–2011 Cairo, Egypt		1840/2616	1.42 (1.11–1.83)*	Crude Odds Ratios	Residence matched, population-based controls	●●●●	●○	○○●○	6
Colon-rectum										
Bener (2010)	CC, 2008–2009 Doha, Qatar		146/282	1.02 (0.62–1.68)	Derived from frequencies (30/146 vs. 57/282)	Matched on sex, race and age (±5 years)	●●○●	○●	○○●	6
Azizi (2015)	CC, 2013–2014 East Azerbaijan, Iran		207/207	1.26 (0.49–3.27)	Derived from frequencies (10/207 vs. 8/207)	Cancer free hospital controls	●●○●	○●	○○●○	5
Liver										
Soliman (2010)	CC, 2007–2009 Tanta, Gharbiah, Egypt		150/150	1.13 (0.62–2.78)	Matched analysis adjusted for viral infection	Hospital healthy visitors matched on sex and age (±5 years)	●●○●	○●	○○●○	5
All cancer deaths										
Wu (2013)	Cohort, 2000–2011 Araihazar, Bangladesh		47/20033	1.30 (0.78–2.18) 2.51 (1.08–5.82)	Adjusted for age, BMI, education	Average follow-up of 7.6 years	●●●●	○●	●●●	8

*CC* case–control study, *NOS* Newcastle Ottawa Scale score

^a^Risk estimate for exclusive waterpipe smokers vs. non-smokers of any type of tobacco

The quality score of the studies assessed using the NOS ranged from 4 to 9. Eleven (39 %) of the 28 studies had a NOS score ≥7 and were considered high-quality studies, respecting generally most of the following criteria: cancer cases were either histological confirmed or identified through hospital records and were representative of all cancer in a defined catchment area over a defined period of time; controls derived from the same population, were selected from the community and had no history of the outcome; analysis was adjusted for other form of smoking if not restricted to exclusive waterpipe smoking and was adjusted for other potential confounders; exposure was assessed by the same method for cases and for controls. Studies with NOS score <7 generally made use of hospital controls, did not provide risk estimates adjusted for other form of tobacco smoking or for additional confounding factors.

**Fig. 2:**
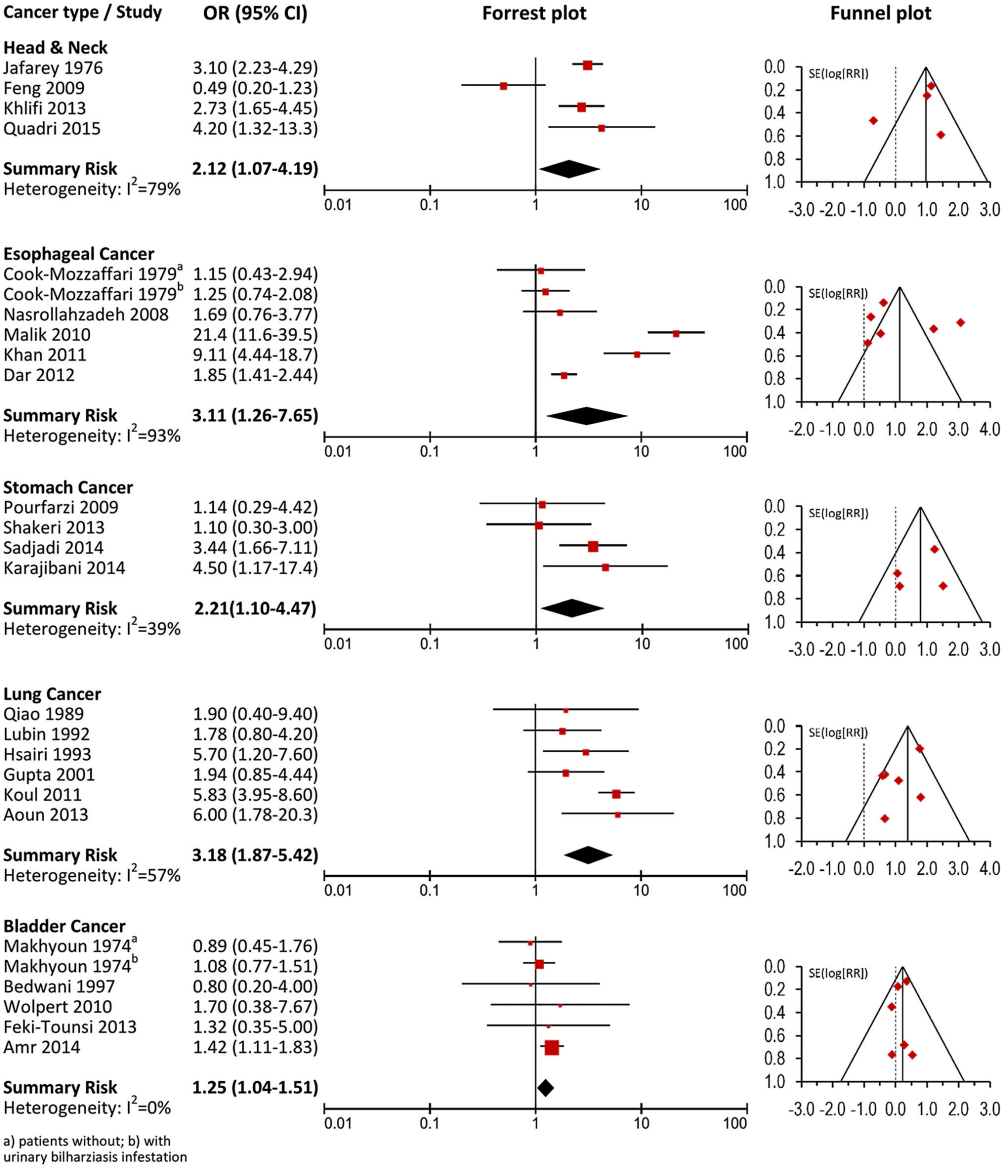
Association between waterpipe smoking and cancer of the head and neck, esophagus, stomach, lung and bladder

Results from the meta-analysis including summary relative risk estimates and 95 % confidence intervals, measures of heterogeneity, forest plots and funnel plots are presented in Table 2 and Fig. 2 for the most studied forms of cancer.

**Table 2:** Summary odds ratios of the association between waterpipe smoking and selected cancer types

Cancer site	All studies			Lower quality studies (NOS <7)			Higher quality studies (NOS ≥7)		
	Studies	OR (95 % CI)	*I* ^2^ (%)	Studies	OR (95 % CI)	*I* ^2^ (%)	Studies	OR (95 % CI)	*I* ^2^ (%)
Head and neck	4	2.12 (1.07–4.19)	79	2	1.40 (0.17–11.4)	88	2	2.97 (2.26–3.90)	0
Esophagus	5	3.11 (1.26–7.65)	93	3	4.11 (0.91–18.6)	95	2	1.84 (1.42–2.38)	0
Stomach	4	2.21 (1.10–4.47)	39	1	4.50 (1.17–17.4)	–	3	1.83 (0.79–4.26)	49
Lung	6	3.18 (1.87–5.42)	57	3	4.13 (1.95–8.72)	65	3	2.22 (1.24–3.97)	0
Bladder	5	1.25 (1.04–1.51)	0	5	1.25 (1.04–1.51)	0	0	–	–

Quality of the studies assessed using the Newcastle Ottawa scale (higher quality studies had a score ≥7)

### Head and neck cancer

Four studies, one from Pakistan (Jafarey et al. 1976), two from Tunisia (Feng et al. 2009; Khlifi et al. 2013) and one from Saudi Arabia (Quadri et al. 2015) reported risks for head and neck cancer with a summary risk of 2.12 (95 % CI 1.07–4.19) (Table 2; Fig. 2). Heterogeneity was present across studies (*I* ^2^ = 79 %), with no evidence of publication bias (Macaskill test *P* = 0.68). In three studies, risk estimates were adjusted for other forms of tobacco smoking (Jafarey et al. 1976; Khlifi et al. 2013; Quadri et al. 2015). The summary risk estimates obtained considering only the two high-quality studies was somewhat higher (OR 2.97; 95 % CI 2.26–3.90).

### Esophageal cancer

Five studies, two from Iran (Cook-Mozaffari et al. 1979; Nasrollahzadeh et al. 2008) and three from the Kashmir valley in India (Malik et al. 2010; Khan et al. 2011; Dar et al. 2012) reported risks for esophageal cancer with a summary risk of 3.11 (95 % CI 1.26–7.65) (Table 2; Fig. 2). Heterogeneity was present across studies (*I* ^2^ = 93 %) with no evidence of publication bias (Macaskill test *P* = 0.36). In one study, the risk estimate was adjusted for other forms of tobacco smoking (Dar et al. 2012); in another the risk was for exclusive waterpipe smoking (Nasrollahzadeh et al. 2008). The summary risk estimate for these two high-quality studies was 1.84 (95 % CI 1.42–2.38).

### Stomach cancer

Four studies, all from Iran (Pourfarzi et al. 2009; Shakeri et al. 2013; Karajibani et al. 2014; Sadjadi et al. 2014), reported risks for stomach cancer with a summary risk of 2.39 (95 % CI 1.43–4.00) with no evidence of heterogeneity (*I* ^2^ = 39 %) or publication bias (Macaskill test *P* = 0.80). (Table 2; Fig. 2). In two studies, risk estimates were adjusted for other forms of tobacco smoking (Shakeri et al. 2013; Sadjadi et al. 2014) and in one the risk was for exclusive waterpipe smoking (Pourfarzi et al. 2009). The summary risk estimate excluding the low-quality study providing only tabular data (Karajibani et al. 2014) was weaker and lost statistical significance (OR 1.83; 95 % CI 0.79–4.26).

### Lung cancer

We identified six case–control studies reporting information on waterpipe smoking and lung cancer risk (Qiao et al. 1989; Lubin et al. 1992; Hsairi et al. 1993; Gupta et al. 2001; Koul et al. 2011; Aoun et al. 2013). Two were from China (Qiao et al. 1989; Lubin et al. 1992), one from Tunisia (Hsairi et al. 1993), two from India (Gupta et al. 2001; Koul et al. 2011), and one from Lebanon (Aoun et al. 2013). The summary risk for the association between waterpipe smoking and lung cancer was 3.18 (95 % CI 1.87–5.42) (Table 2; Fig. 2) with moderate heterogeneity across studies (*I* ^2^ = 57 %) but no evidence of publication bias (Macaskill test *P* = 0.54). The odds ratios reported in each individual study ranged from 1.78 (95 % CI 0.80–4.20) (Lubin et al. 1992) to 6.00 (95 % CI 1.78–20.3) (Aoun et al. 2013). Only one study provided risk estimates adjusted for cigarette use (Hsairi et al. 1993), and three studies reported lung cancer risk associated with exclusive waterpipe smoking (Qiao et al. 1989; Lubin et al. 1992; Koul et al. 2011). The summary risk of lung cancer considering the three high-quality studies (Qiao et al. 1989; Lubin et al. 1992; Hsairi et al. 1993) was 2.22 (95 % CI 1.24–3.97) with no evidence of heterogeneity (*I* ^2^ = 0 %).

### Bladder cancer

Five studies, four from Egypt (Makhyoun 1974; Bedwani et al. 1997; Wolpert et al. 2010; Amr et al. 2014) and one from Tunisia (Feki-Tounsi et al. 2013) reported on the association between waterpipe smoking and bladder cancer (Table 2; Fig. 2). The summary risk for all studies was 1.25 (95 % CI 1.05–1.51) with no evidence of heterogeneity across studies (*I* ^2^ = 0 %), but none were classified as of high-quality. Examination of the funnel pot indicated no evidence of publication bias although the test proposed by Macaskill et al. was statistically significant (*P* = 0.03), being largely influenced by estimates of the largest study (Amr et al. 2014). Excluding this study, the summary risk was 1.06 (95 % CI 0.80–1.41) with no evidence of publication bias (Macaskill test *P* = 0.94).

### Other forms of cancers

Four studies reported on the association between waterpipe smoking and other various form of cancer (Table 1) for which it was not possible to calculate summary risk estimates due to the limited number of studies for each single cancer site. Two studies were focused on colorectal cancer: one from Qatar (Bener et al. 2010) and one from Iran (Azizi et al. 2015) with non-significantly increased risks of 1.02 (95 % CI 0.62–1.68) and 1.26 (95 % CI 0.49–3.27), respectively. One study from Egypt reported a risk of 1.13 (95 % CI 0.62–2.78) for liver cancer (Soliman et al. 2010). Finally a high-quality study from Bangladesh looked at all forms of cancer mortality in a cohort of 20,033 individuals and reported a significant risk of cancer death equal to 2.51 (95 % CI 1.08–5.82) for those who were current waterpipe smokers at the time of interview (Wu et al. 2013).

## Discussion

In this report we have used previously reported data on the relationship between waterpipe smoking and neoplasms in a meta-analytic approach to define the association between this type of tobacco exposure and cancer. We found 28 published studies with sufficient exposure data and statistical information to allow us to calculate summary odds ratios for the risk of several cancers known to be linked to tobacco exposure. The overall report is based upon 8,714 cancer cases and 35,746 controls, making this study more comprehensive than previously published reports (Akl et al. 2010; Chaouachi 2006).

In a previous meta-analysis Akl et al. (2010) found that waterpipe exposure resulted in a significant excess of lung cancer, but not to an increased risk of upper digestive tract cancer or bladder cancer. With a larger sample size including additional studies, we now find that waterpipe smoking is related not only to lung cancer but also to cancer of the head and neck, esophagus, stomach and to bladder cancer.

There was considerable inter-study heterogeneity in the overall estimates of risk for lung cancer, head and neck cancer, and for esophageal cancer. This may be related to variation in definition of waterpipe exposure (yes/no, ever/never) or the use of different control groups—either non-waterpipe smokers, or non-smokers of any type of tobacco. Another source of heterogeneity is that the reports originated from 10 different regions with different smoking patterns, and where waterpipe smokers used different types of equipment. A final source of variation could be related to temporal-related changes in smoking occurring over the 40 years spanning the publication of these reports. In fact, waterpipe is becoming the most popular form of tobacco smoking among youth in the Middle East, and is gaining popularity elsewhere (Maziak et al. 2015).

Publication bias is another concern since statistically significant or important associations are more likely to be published and reported in the titles or abstract of the papers. To limit such bias, we used different databases for the identification of relevant studies. Sixteen studies were identified using PubMed searching for keywords in the title, abstract or Medical Subject Headings (MeSH) indexes and 12 from other sources, including references or citations of major papers on the topic. We assessed publication bias visually inspecting asymmetry of the funnel plot and using the test proposed by Macaskill et al. (2001). We found no evidence of publication bias for head and neck, esophageal, stomach or lung cancer, but the number of studies at each site was limited making assessment of bias uncertain. Potential publication bias was present only for bladder cancer and could be attributed to the largest study. After exclusion of this report in a sensitivity analysis no evidence of publication bias remains.

Unfortunately, there were not enough reports to assess geographical patterns of the cancer site-specific risk associated with waterpipe smoking. For various reasons (local research interest, high incidence of a specific type of cancer in a region, publication of a previous report requiring confirmation,…) related studies were often conducted in similar areas: all studies reporting on stomach cancer were conducted in Iran, those reporting on esophageal cancer risk were conducted either in the Kashmir valley or in Iran; four of the five studies reporting on bladder cancer risk were conducted in Egypt.

This study clearly shows that waterpipe smoking increases the risk for several common cancers. For esophageal and stomach cancer, the risk resembles the risk associated with conventional cigarette smoking. If cigarette smokers switch to waterpipe smoking, the proportion of tobacco-related cancer in these organs will remain the same (Engel et al. 2003).

Using pack-years, it is possible to calculate a dose response for the risk of cancer associated with cigarette smoking. Despite the availability of software for comparing waterpipe exposure to cigarette exposure (Masters et al. 2015), calculating a similar dose response for waterpipe exposure is more difficult and data on a dose-related cancer risk has been infrequent in previous reports. In one report looking at a dose response for waterpipe smoking and esophageal cancer, with never users as the comparison group, those who smoked 1–139, 140–240 and more than 240 hookah-years had respective risks of 1.12 (95 % CI 0.77–1.64), 1.54 (95 % CI 1.05–2.26) and 3.62 (95 % CI 2.50–5.23) to develop esophageal cancer (*P* for trend <0.0001) (Dar et al. 2012).

Reports on exposure to cancer-related carcinogens in waterpipe smoke support the case–control data used in this meta-analysis. These reports disprove the widely held belief that filtering tobacco smoke through a container of water removes carcinogens derived from burning tobacco (Al Ali et al. 2015; Jacob et al. 2013; Radwan et al. 2013; Daher et al. 2010; Sajid et al. 2007; Jones et al. 1990).

Just as second-hand cigarette smoke is a known risk factor for cancer, the high levels of side-stream smoke in waterpipe cafes can lead to increased levels of tobacco-related nitrosamines in both smokers and non-smokers exposed to the ambient air of waterpipe cafes posing a risk to both smokers and non-smokers exposed to this environment. (Radwan et al. 2013; Moon et al. 2015; Al Mulla et al. 2015).

This report has several weaknesses. In comparison to the thousands of articles looking at the cigarette-associated cancer risk, there were very few studies available for this meta-analysis; many were small studies with only a limited number of cancer patients in each report. In addition, it is probable that in some reports waterpipe smokers included current or previous cigarette smokers, which would bias the results. Also, for the comparison control population, most of the studies used hospital controls or hospital visitors, rather than a random sample of the population. The limitations of the available data point out the need for larger carefully designed studies in well-defined populations.

In summary, this report supports and quantifies the risk of cancer in waterpipe smokers. In general, the types of cancers reported in waterpipe smokers are similar to the types of tumors observed in cigarette smokers but the number and quality of studies available for a definite assessment is very limited. Results from high-quality studies, however, indicate significant increased risk for cancer of the head and neck, esophagus and lung. More high-quality studies would be necessary to properly assess the risk for other forms of cancers. Controlling the impending epidemic of waterpipe smoking will require the combined efforts of health educators, legislators, public health officials, and research scientists.
